# Strong Precipitation and Human Activity Spur Rapid Nitrate Deposition in Estuarine Delta: Multi-Isotope and Auxiliary Data Evidence

**DOI:** 10.3390/ijerph18126221

**Published:** 2021-06-08

**Authors:** Hanyou Xie, Chong Huang, Jing Li, Yitao Zhang, Xiangbo Xu, Deyao Liu, Zhu Ouyang

**Affiliations:** 1 Key Laboratory of Ecosystem Network Observation and Modeling, Institute of Geographic Sciences and Natural Resources Research, Chinese Academy of Sciences, Beijing 100101, China; xiehy.18s@igsnrr.ac.cn (H.X.); zhangyt@igsnrr.ac.cn (Y.Z.); ydxu.ccap@igsnrr.ac.cn (X.X.); liudeyao20@mails.ucas.ac.cn (D.L.); 2 College of Resource and Environment, University of Chinese Academy of Sciences, Beijing 100049, China; 3 State Key Laboratory of Resources and Environmental Information System, Institute of Geographic Sciences and Natural Resources Research, Chinese Academy of Sciences, Beijing 100101, China; huangch@igsnrr.ac.cn; 4 UN Environment-International Ecosystem Management Partnership (UNEP-IEMP), Beijing 100101, China; 5 Yellow River Delta Modern Agricultural Engineering Laboratory, Institute of Geographic Sciences and Natural Resources Research, Chinese Academy of Sciences, Beijing 100101, China; ouyz@igsnrr.ac.cn

**Keywords:** nitrate N transport, multi-stable isotopes, auxiliary geographic information, heavy rainfall-runoff, Yellow River Delta

## Abstract

The intensive development of the Yellow River Delta has caused huge transportation of non-point pollutants into the Bohai Sea through source river estuaries and thus poses a considerable threat to eco-environmental security in the region. Long-term irrigation in the Yellow River basin, with occasional heavy rainfall and the related effects of ensuring hydrological processes and human activities in terms of nitrate N transport via surface water systems, is unclear. Using stable isotope (δ^2^H-H_2_O and δ^18^O-H_2_O, δ^15^N-NO_3_^−^ and δ^18^O-NO_3_^−^) and auxiliary geographic data, the ISO source model was run to quantitatively analyze the supply relationship of river systems and the rapid change in the spatial pattern of nitrate N due to heavy rainfall in the estuarine delta. This analysis made clear the dominant contribution of agricultural activities and urbanization to NO_3_^−^-N emission, on which basis refined management measures were proposed to deal with NO_3_^−^ in surface water from the “source-process”. The results of the study show that: (1) The relationship of surface water replenishment in the Yellow River Delta was affected not only by rainfall, irrigation, and other water conservancy measures but also the proportion of water from Yellow River flow declined from the source to estuary; (2) To a certain extent, rainfall diluted the concentration of nitrate N in the river and increased instantaneous flux of nitrate N into the sea, where nitrate N flux continuously increased from upstream to downstream; (3) The rapid deposition of nitrate in the estuary delta was driven by heavy rainfall and human activities such as excessive use of nitrogen fertilizers, rapid urbanization, and livestock waste discharge, and; (4) Scientific measures were needed to realize the interactive effect of the output of non-point source pollutants and the carrying and absorption capacity of coastal fragile ecosystems of the exogenous inputs.

## 1. Introduction

Rivers are a key channel for nitrate transport between terrestrial surfaces and the oceans. Eutrophication of rivers and coastal waters due to nitrate load is widely studied [1]. Nearly 500 million people live in and around global delta areas such as the Nile, Indus, Po, and Yellow River Delta, with high population density and increasing intensity of agricultural activities [2]. Nitrogen emission at river outlets from the land into the sea has increased by 90% in the last century. Although this has resulted in the deterioration of water quality in coastal areas, nitrogen concentrations in rivers will continue to rise, especially in agricultural catchments [3]. The maximum allowable nitrate concentration in a river is 50 mg/L [4]. In China, 27% of coastal basins do not meet the class IV water quality standard [5], and over 51% of the sea areas are experiencing eutrophication, with the main pollutant being inorganic nitrogen [6].

The prerequisite for controlling and managing nitrate pollution is the determination of the source of pollution [7]. Studies show that the sources of nitrate in surface water are many, including urban and industrial sewage, agricultural fertilizers, human and livestock manure, atmospheric precipitation, and soil organic nitrogen [8]. Nitrate pollution in water is affected by geographical location, land use type, climate, and hydrological characteristics [9]. Nitrate N concentration in surface waters in the Pearl River Delta is 0.85–5.89 mg/L. The urban expansion increases NO_3_^−^-N concentration and sewage is the main source in this case [10]. Cultivated land area is related to nitrate concentration in surface water [11]. Additionally, the development and utilization of agriculture can increase nitrate N concentration in water bodies [12]. Furthermore, nitrate widely occurs in areas with intensive human activity [13].

Intensive agriculture and sudden precipitations are the main factors driving nitrogen distribution in space and time, including in surface water bodies [14,15]. Traditionally, farmers use nitrogen fertilizer on surface soil before heavy rains or flood irrigation. However, the amount of drainage and leaching of nitrate increases with increasing irrigation amount. Studies show that nitrogen loss in the 0–90 cm soil layer in the North China Plain is in the neighborhood of 63% [16]. During periods of intensive rainfall, surface erosion can enhance the amount of nitrate in runoff water [17], The contribution rate of chemical fertilizer, soil organic nitrogen, and human and animal manure to nitrate in river systems is more than 93% [18].

The ISOsource model was developed by the US EPA back in 2003 and has been widely used in the analyses of the water cycle and nitrate tracing [19,20,21]. Built on mass conservation of stable isotopes and multi-source linear mixing, the model is used to estimate the contribution ratio of different material sources. Stable isotopes of water (δ^2^H-H_2_O and δ^18^O-H_2_O) and nitrate (δ^15^N-NO_3_ and δ^18^O-NO_3_) are powerful tools used in source determination [9,22].

The Yellow River Delta is the youngest estuary Delta in the world and has widespread saline land because of its low precipitation evaporation ratio, seawater impact, etc. [23]. In the effort to increase the development and utilization of this area, farmers increase fertilizer use in crop production. This has improved the generally poor soil structure and low soil fertility, making the area one of the regions with the highest nitrogen application in China [24]. Excess nitrogen enters into the water body through runoff or infiltration and participates in the water cycle [25]. Precipitation in the area is highest in the July-August summer period, which is short but acute. Irrigation water in the region is mainly from the Yellow River and flood irrigation is usually preferred. Some 840,000 tons of pollutants enter the Bohai sea annually, predominantly inorganic nitrogen [26]. In the area, the average concentration of nitrate in groundwater is 30 mg/L [27]. The nitrate N is 46.7% of that in the reach of the Yellow River that spans the irrigation area, exceeding the WHO drinking water standard values for NO_3_^−^N [7]. Although the rains coincide with the main period of nitrogen transport, little work has focused on nitrate dynamics before and after rainfall events in the low plain estuarine delta [28].

To determine nitrate dynamics in surface rivers in the estuarine delta region during rainfall events, a large-scale sampling was conducted in the Yellow River Delta of China. With the help of the dual nitrate isotope method and auxiliary geographic data, a rapid nitrate N transport in the estuary delta (driven by heavy rainfalls and human activities) is quantitatively tracked by combining recharge characteristics of surface water and nitrate source. The purpose of this study was: (1) to understand the relationship between nitrate dynamics and discharge in estuarine delta rivers; (2) identify nitrate sources and estimate their contributions, and (3) explore the main factors controlling nitrate dynamics in heavy rainfall events. The results will provide a scientific basis for understanding the source, transport process, flux, and emission control of nitrate N in the study area.

## 2. Materials and Methods

### 2.1. Introduction to the Study Area

The study area is located at the estuary of the Yellow River, which spans from the south of the Yellow River to the north of the tributary river (37.26–37.83 N, 118.21–119.29 E) with an area of 2841 km^2^. The area has a semi-humid temperate continental monsoon climate, with precipitation of 673.9 mm in 2019. The rainy season is from July to September and groundwater level fluctuates from 1.0–2.5 m. There are eight main rivers in the study area, including Yellow Rive Farm Ditch, Xiaodao, Zhangzhen, Yongfeng, Yihong, Guangpu, Guangli, and Zhimai Rivers. While Guangpu and Yihong Rives are tributaries of Zhimai and Guangli River, respectively. Xiaodao and Zhangzhen share one estuary. The Yellow River irrigation project in the irrigation district in the lower reach (Haihe River Basin) started in 1855. Long-term irrigation has changed the distribution of the regional hydrological and water resources along with the spatio-temporal distribution of NO_3_^−^-N in the basin.

The study area is a key agricultural production base in China (http://www.stats.gov.cn/, accessed on 19 September 2020). The cultivated area is 280,000 ha and the main crops include wheat, corn, cotton, rice, and soybean. The annual reserves of heads of cattle, sheep, and pig are 240,000, 2,086,000, and 1,197,000, respectively.

### 2.2. Sample Collection and Analysis

**Figure 1:**
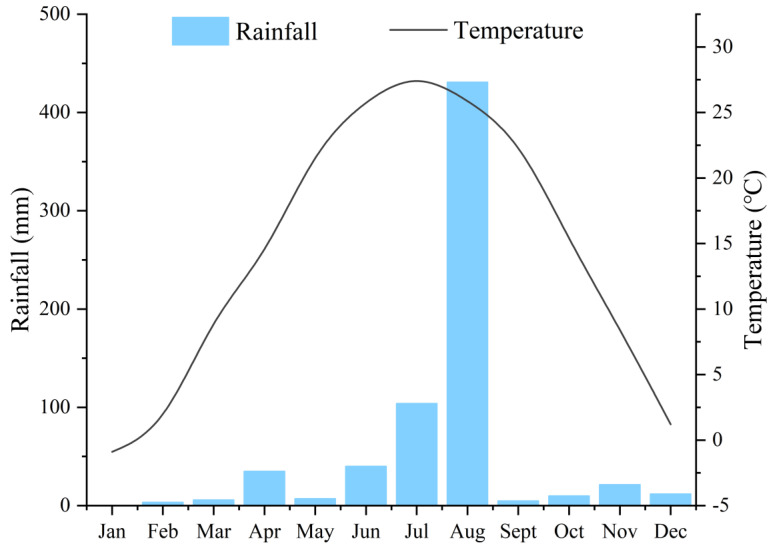
Monthly rainfall and average temperature.

**Figure 2:**
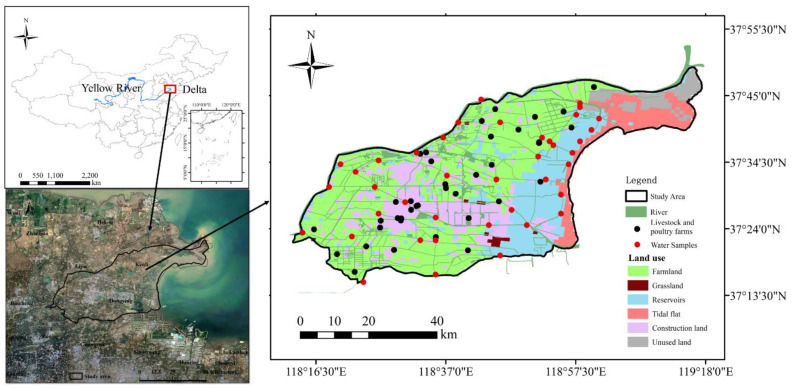
Study area location and sampling points.

The rainy season in 2019 started on 23 July and ended on 27 August, lasting for 36 days. The heaviest rainfall was on August 20 and was 322.0 mm; accounting for 47.8% of the annual rainfall (Figure 1). In this study, 40 sampling points were set up in the main rivers, tributaries and the confluences and surface water samples were collected before and after heavy rains (Figure 2). The bank-to-bank width of the water surface was measured using a hand-held laser rangefinder (SPI600) at each sampling point. The water depth and flow velocity were measured using a portable doppler ultrasonic flowmeter (DX-LSX-2) at 1/6, 1/3, and 1/2 of the river width, and the river shape (rectangle, trapezoid, or arc) was recorded. Some 500 mL of the sample was collected in a plastic bottle, moistened with ultrapure water three times, air-dried, and stored at −20 °C for the determination of other indicators. Rainwater samples were collected from March to October 2020. NO_3_^−^-N concentration of the water was determined using ultraviolet spectrophotometry (UV-2700). Firstly, the standard nitrate-nitrogen solution of 0 mL, 0.5 mL, 1.0 mL, 1.5 mL, 2.0 mL, 2.5 mL, 5.0 mL, and 7.5 mL was diluted to 50.0 mL with pure water, and 1.0 mL hydrochloric acid solution was added and mixed evenly. Secondly, the absorbance was measured with pure water at 220 nm and 275 nm. The absorbance at 220 nm (A220) was subtracted twice at 275 nm (A275) to obtain the linear equation for the mass concentration of nitrate nitrogen. Finally, 50 mL of water sample was taken and acidified with 1.0 mL of hydrochloric acid solution. The absorbance was measured at 220 nm and 275 nm, respectively. The nitrate-nitrogen content of the water sample was obtained according to the regression equation. [29]. The water sample was filtered in a 0.45 μm micro-porous filter and stored in brown injection bottles for cold storage. It was then sent to the Institute of Geographical Sciences and Natural Resources Research of the Chinese Academy of Sciences for stable isotope analysis (δD-H_2_O and δ^18^O-H_2_O); with a liquid water isotope analyzer (LGR DLT-100) as the detection instrument. The organization for the detection of stable isotopes of nitrate (δ^15^N- NO_3_^−^ and δ^18^O- NO_3_^−^) is the Institute of Environment and Sustainable Development in Agriculture, Chinese Academy of Agricultural Sciences. The denitrifying bacteria method was used and the detection instrument model was the Delta-Plus Precon. The denitrifying bacteria method is to reduce NO_3_^−^ in the water body to N_2_O, and then extract and purify N_2_O before measuring the nitrogen and oxygen isotope, the nitrogen and oxygen isotope of NO_3_^−^ in the sample can be determined [30].

The measured stable isotope content was expressed as the thousandth difference relative to the standard substance; expressed as δ:

(1) $$\delta_{\mathrm{sample}}=\left(\frac{R_{\mathrm{sample}}}{R_{\mathrm{standard}}}-1\right)\times 1000‰$$

Average ocean water (V-SMOW) and air nitrogen (N_2_) were used as the standards for D-^18^O and δ^15^N-NO_3_^−^, respectively [31].

### 2.3. ISOsource Model

In this study, the sources of surface water were divided into seawater, precipitation, Yellow River, and groundwater [32]. Then the sources of nitrate included precipitation, industrial fertilizer, human/livestock manure, and soil organic nitrogen as showed in Table 1 [33,34]. The mass conservation model was constructed by inputting isotope values from different sources as follows:

(2) $$\delta_{m}=f_{a}\delta_{a}+f_{b}\delta_{b}+f_{c}\delta_{c}+\cdots +f_{n}\delta_{n}$$

(3) $$\lambda_{m}=f_{a}\lambda_{a}+f_{b}\lambda_{b}+f_{c}\lambda_{c}+\cdots +f_{n}\lambda_{n}$$

(4) $$1=f_{a}+f_{b}+f_{c}+\cdots +f_{n}$$

where *f_n_* is the proportion of different surface water or nitrate sources; $\delta_{n}$ is D or ^15^N from different sources; *λ_n_* is ^18^O from different sources, and $\delta_{m}$ and *λ_m_* are D-^18^O or ^15^N-^18^O isotope values in the water sample.

Then the tolerance and incremental parameters of the model were set and the probability distribution of the percentage of different sources in the sample was calculated by the iterative method. All possible percent combinations of different sources (and 100%) were calculated based on the following equation:

(5) $$\mathrm{Number}\;\mathrm{of}\;\mathrm{combinations}\;=\left(\frac{\frac{100}{i}}{s-1}+1\right)=\frac{\left[\left(\frac{100}{i}\right)+\left(s-1\right)\right]!}{\left(\frac{100}{i}\right)!\left(s-1\right)!}$$

In Equation (5), *i* is the incremental parameter and *s* is the number of isotopic sources. The first mock exam can test all possible combinations of potential contributions from each source. When the isotopic average of the isotopic values of different sources was less than 0.1 per thousand, the isotope was not considered as a possible solution [19,35]. The calculation process was carried out in ISOsource software, downloaded from the United States Environmental Protection Agency (https://www.epa.gov/eco-research/, accessed on 7 July 2019). Specific steps for data processing are: 1—running the IsoSource and entering the source and sample point isotope values; 2—imposing additional constraints and running the calculation; 3—reading the “.out” file and getting the report distribution of constrained solutions; 4—data analysis was conducted using the Microsoft Excel 2019.

### 2.4. Water System and Land Use Data

The water network and land use data were from the Resource and Environment Science and Data Center, IGSNRR, Chinese Academy of Sciences (http://www.resdc.cn/, accessed on 18 October 2019). Based on the shape of the river, univariate quadratic, rectangular or trapezoidal equations were constructed using water depth at 1/6, 1/3, and 1/2 of water surface width and river surface width to calculate the river section area. The equation instantaneous nitrate N flux is [18]:

(6) $$N_{f}=S\times V*C$$

In Equation (6), N*_f_* is instantaneous nitrate N flux (g/s); S is cross-sectional area (m^2^); V is average horizontal velocity (m/s); and C is nitrate N concentration (mg/L).

## 3. Results and Discussion

### 3.1. Heavy Rains Changed the Relationship of Surface Water Replenishment

The relationship between surface water replenishment in the Yellow River Delta is affected by meteorological factors and irrigation measures such as irrigation from the Yellow River [36]. The surface water before rains is mainly from the Yellow River water and groundwater. Then water replenishment in the delta is from the Yellow River water and precipitation. The proportion of recharge from the Yellow River into the other rivers in the study area declined in trend. This was because water diverted from the Yellow River was consumed in the surrounding farmlands or went as recharge to groundwater [37]. When water was diverted from the Yellow River into other rivers before the rains, replenishment was from groundwater in the middle reaches. This led to seawater intrusion in the downstream areas, accounting for a relatively high proportion of seawater [38,39]. The mode of replenishment after the rains changed was dominated by precipitation in the middle and lower reaches of the Yellow River Delta. Guangli and Zhimai Rivers are the two largest rivers in the study area. The proportion of Yellow River discharging into the two rivers before precipitation was relatively high. This was because the rivers have large drainage areas, large amounts of diverted water from the Yellow River, and strong replenishment for surface water. However, the rivers had the highest precipitation replenishment ratio after the rains because they received large amounts of precipitation upstream [10,40].

**Figure 3:**
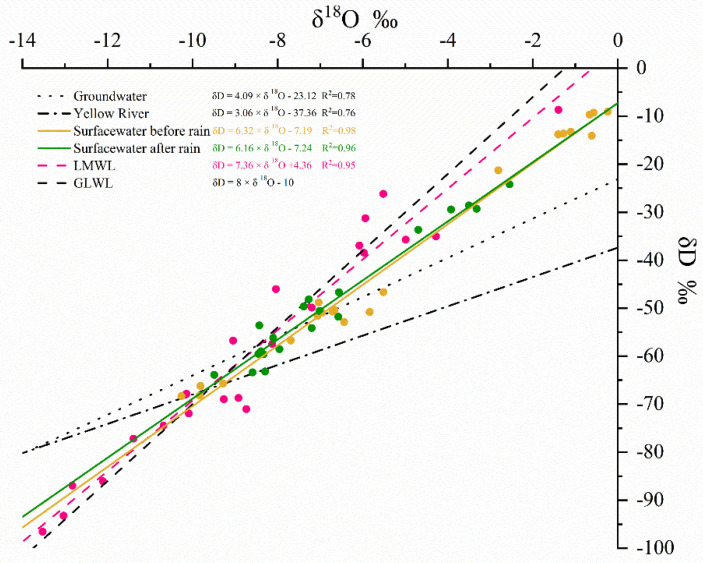
The lines of fit distributions between δD-H_2_O and δ^18^O-H_2_O in the study area.

Evaporation before the rain was strong and less precipitation replenished surface water, resulting in isotopic enrichment. The range of surface water δD in the study area was −9.09–−68.48‰ (average of −41.60‰) and that of δ^18^O was −10.26–−0.24‰ (average of −5.21‰). Driven by rainfall, stable isotopes in the waters after the rain decreased; with δD range of −24.19–−72.44‰ (average of −54.48‰) and δ^18^O range of −2.55–−10.16‰ (average of −7.68‰) (Figure 3). Li et al. [32] reported similar results in terms of the change in trend in isotopes surface waters in the region during low and high water periods. The fit for the relationship between δD and δ^18^O also supported the above results. The slope before the rains was lower than that after the rains, indicating that the evaporation effect before the rains was stronger. δD and δ^18^O values in surface water were scattered between the local atmospheric precipitation line (LMWL), groundwater, or Yellow River water oblique line. This further indicated that surface water availability in the area was affected by atmospheric precipitation, groundwater, and water diversion from the Yellow River. The slope of LMWL (7.36) was slightly smaller than the slope of the global atmospheric precipitation line GMWL (8.0) [35], similar to that reported by Du et al. [41] for Laizhou Bay.

**Figure 4:**
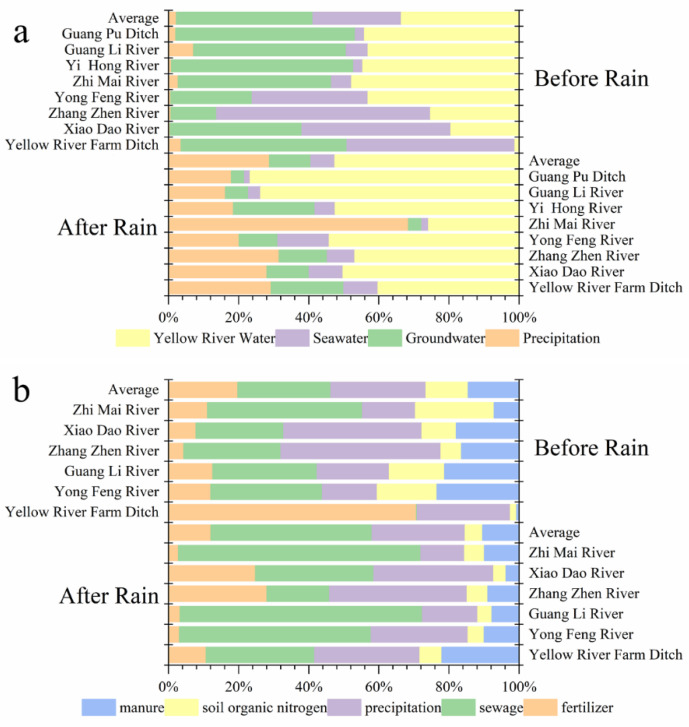
The average recharge ratios of river water (**a**) and nitrate sources (**b**) in the study area.

The ISOsource model was established based on δD-^18^O dual-isotope and the source and contribution ratios of surface water analyzed (Figure 4a). Before the rains, surface water was mainly recharged by groundwater (39.0%) and the Yellow River (33.6%). The proportions of precipitation (2.3%) and seawater (25.2%) recharge were relatively low. The water sources of different rivers were quite different. Water replenishment in the study area after the rains was dominated by water from the Yellow River (52.5%) and precipitation (28.8%), and the average seawater replenishment ratio was 6.8%. Guangli River had the highest proportion of water from the Yellow River (73.7%), and precipitation was the main water source for the Zhimai River (68.5%).

**Figure 5:**
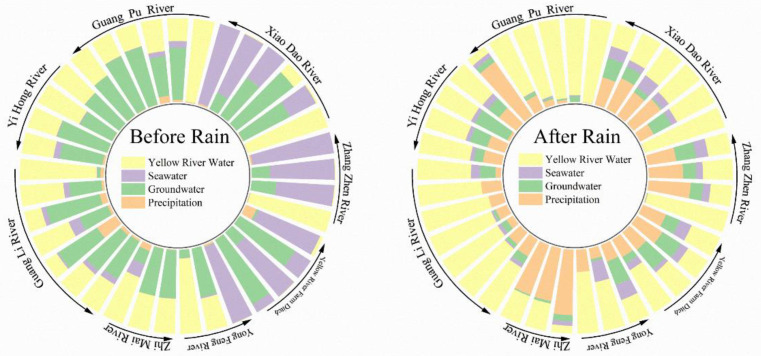
Changes in recharge from upstream to downstream regions in the study area before and after the rains.

The water replenishment ratio of each river (from upstream to downstream) is plotted in Figure 5. While the proportion of water recharge from the Yellow River (starting from the source to the estuary) decreased in trend before the rains, that from groundwater and seawater increased. The proportion of replenishment from the Yellow River into the Xiaodao River at the source was (99.7%) and that from seawater was (0%). Then the respective replenishment proportions of the mouth into the sea were 0% and 98.0%. The ratio of groundwater recharge to Zhangzhen, Yongfeng, and Guangpu Rivers initially increased in trend and then decreased. The proportion of the Zhimai River that was from the Yellow River dropped from 59.0% to 41.8% in the upper reaches, and that of Guangli River dropped from 91.5% to 38.5%. While recharge from the upper reaches into the lower reaches of the water from the Yellow River continued to drop after the rains, precipitation recharge increased. Recharge from the Yellow River into the Xiaodao River dropped from 99.5% to 27.5%, but precipitation recharge increased from 0.5% to 34.0%. Recharge from precipitation and the Yellow River into the middle and lower reaches of the Zhangzhen River was respectively 34.0–50.3% and 26.0–35.5%. Recharge from the Yellow River to the Yongfeng River and Yellow River Farm Ditch decreased respectively by 43.5% and 11.0% from the upstream to the downstream. The proportion of precipitation in the Zhimai River increased from 63.5% to 78.0%.

### 3.2. Heavy Rains Changed the Nitrate Distribution and Promoted Its Rapid Output

Rainfall increases instantaneous nitrate N flux in surface water. In this light, the Yongfeng River had the highest increase in nitrogen flux (685.9%), followed by the Xiaodao and Zhangzhen Rivers (298.1%), and Guangli River had the lowest increase (3.8%). Nitrate N flux in the Zhimai River increased by 102.5%. Nitrate N flux into the sea before the rains was 499.16 and after was 862.59 g/s, a combined increase of 72.8%. Based on the results in Table 2, nitrate N flux from the rivers into the sea after the rains was positively correlated within the basin area. The flux in the branch river (493.51 g/s) > Guangli River (179.05 g/s) > Xiaodao River and Zhangzhen River (85.4 g/s) > Yellow River Farm Ditch (49.33 g/s) indicated that rivers with larger basin areas had greater runoff, significantly influencing nitrate N flux into the river [42].

**Figure 6:**
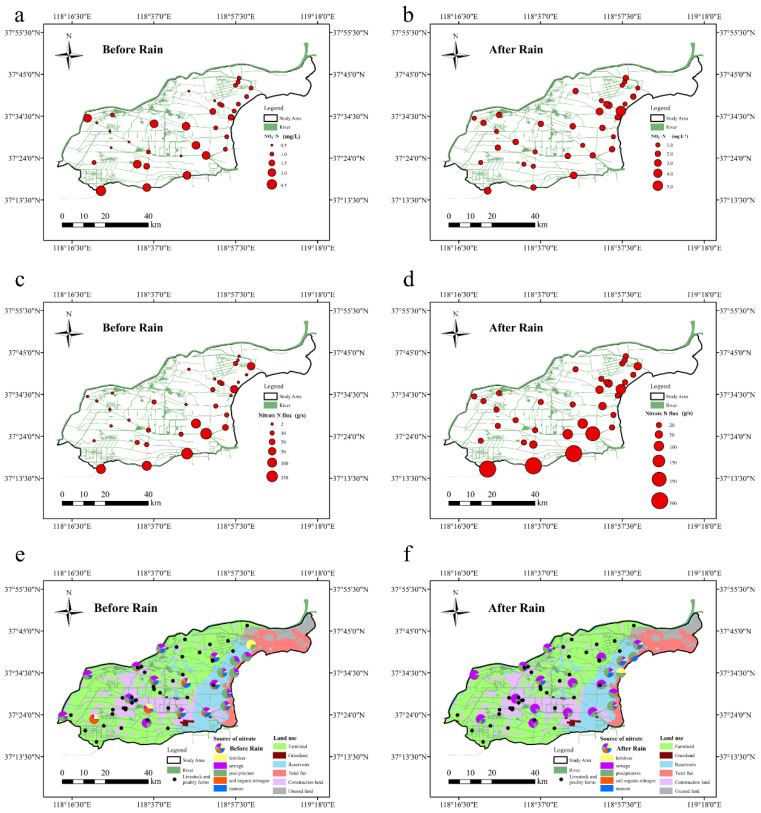
Spatial characteristics of surface water nitrate N concentrations (**a**,**b**), instantaneous fluxes (**c**,**d**), and source contribution ratios (**e**,**f**) before and after rains in the study area.

The concentration of NO_3_^−^-N before the rains was 0.10–4.06 mg/L, with an average of 1.37 mg/L (Figure 6). The Zhimai River had the highest concentration (3.33 mg/L) and Yellow River Farm Ditch had the lowest (0.70 mg/L). The average nitrate N concentrations in the Xiaodao River and Guangli River were 0.95 and 0.91 mg/L, respectively. The average NO_3_^−^-N concentration in surface waters of Zhangzhen, Yongfeng, Yihong, and Guangpu Rivers was 1.47–1.93 mg/L. After rains, nitrate N concentration was 0.57–4.86 mg/L, with an average of 1.47 mg/L. The average nitrate N concentration in Xiaodao River was 2.16 mg/L, an increase of 128.1% over that before rains. NO_3_^−^-N concentration in the branch canal of Yellow River Farm Ditch and Guangli River was 0.61–1.40 mg/L, and an increase of 34.4% and 10.3%, respectively. Based on Figure 5, the ratio of Yellow River water and precipitation recharge for the three rivers before and after rains increased respectively by 30.7–39.1% and 9.1–27.6%. The average nitrate N concentration in Yellow River water before the rains was 2.39 mg/L and that after was 2.68 mg/L, much higher than the average concentration for the three rivers. This suggested that the Yellow River water was one of the main nitrate N sources in the basin.

NO_3_^−^-N concentrations before and after rains at the estuary were 0.93 and 0.96 mg/L, lower than the average concentration in the study area respectively by 31.9 and 35.0%. Compared with the nitrate N content of the Bohai Sea (0.06–1.61 mg/L), it was at medium level and similar to nitrate concentration in the offshore (0.99 mg/L) region [43]. After rains, nitrate N in Yongfeng (1.28–2.80 mg/L), Yihong (0.84–1.54 mg/L), Guangpu (0.73–2.05 mg/L), and Zhimai (1.22–2.50 mg/L) Rivers decreased respectively by 6.6, 23.0, 36.4, and 42.2%. Then precipitation recharge ratio in the four rivers increased by 15.9–65.7%. Guo et al. [6] showed that the concentration of NO_3_^−^-N in the dry season in the study area was 0.55–3.77 mg/L and that in the rainy season it was 0.47–2.65 mg/L. This was due to the heavy rains, which diluted nitrate N concentration in the surface water systems [42,44].

Nitrate N flux increased from upstream to downstream. Before rains, nitrate N flux at the source and estuary of the Xiaodao River were respectively 0.01 and 21.45 g/s. For Yellow River Farm Ditch it increased from 0.19 to 34.18 g/s. It nearly doubled from 1.96 g/s in the Yongfeng River. The fluxes at the source and estuary of the Zhimai River were 60.12 and 243.76 g/s, respectively. Nitrate N flux into the sea before the rains was 0.05–243.76 g/s and into the sea from the Zhimai River (243.76 g/s) was the highest, followed by Guangli River (172.50 g/s). Nitrate N flux into the sea from Yellow River Farm Ditch, Xiaodao, Zhangzhen, and Yongfeng Rivers were respectively 34.18, 21.45, and 3.87 g/s. After rains, nitrate N flux in the Xiaodao River increased from 2.57 to 85.40 g/s. While nitrate N flux at the sources of the Guangli and Zhimai Rivers were respectively 1.69 and 362.73 g/s, those into the sea were 179.06 and 493.54 g/s. Nitrate N flux into the Guangpu River was 0.23–44.63 g/s. Then the flux from the Yongfeng River into the sea was 30.38 g/s, 4.13 times higher than that in the upstream region.

### 3.3. Human Activities Dominate the Source of Surface Water Nitrate in the Yellow River Delta

In this study, the values of δ^15^N-NO_3_^−^ and δ^18^O-NO_3_^−^ in different rivers are shown in Table 2. The range of δ^15^N in the study area was −1.52–13.24‰, and that of δ^18^O was −11.70–28.68‰. Double isotopes were accurately traced from the nitrate in the collected water samples [45]. The IsoSource model data analysis showed that the dominant sources of nitrate in the study area included human activities such as the use of fertilizers in agriculture, industrial and residential sewage, and human and animal manure. The total contribution was 60.8% before and 68.4% after the rains (Figure 4b). The sources of nitrate before the rains were mainly human/animal manure/sewage, with an average proportion of 41.0% and that for chemical fertilizers of 19.8%. After the rains, the proportion of nitrate source from sewage (45.9%) in the study area was highest; 19.5% higher than before the rains, similar to the results of Duan et al. [46]. The contribution rates of chemical fertilizers (12.1%) and human/animal manure (10.4%) dropped respectively by 7.7% and 4.1%. Human activities affected the sources of nitrate in the study area.

Irrigation from the Yellow River affected the source ratio of nitrate in surface water in the upper reaches of the study area [47]. Analysis of the sources of nitrate in surface water in the upper reaches of the Yongfeng, Guangli, and Zhimai Rivers and their tributaries showed a stable proportion of nitrate sources. The proportion of chemical fertilizers was 4.5–7.2%, that of sewage 34.8–48.0%, and of precipitation 22.5–27.0%. The contribution rates of soil organic nitrogen and human/animal manure were respectively 6.5–12.8 and 12.0–24.8%. This was probably because the sources of water supply upstream were similar. The R^2^ fit of the ratio of nitrate from sewage sources in the Guangli River to that of the water recharge from the Yellow River before and after the rains were 0.90 and 0.71, respectively. Yue et al. [33] noted that the main contributors of nitrate in the Yellow River were human/animal manure and sewage.

Fertilization applications on farmlands increased the rate of contribution of nitrate from fertilizer sources to surface water nitrate. The proportion of fertilizer sources in Yellow River Farm Ditch was 70.8%, caused by fertilization of paddy fields in the area [48] After the rains, the proportion of nitrate from upstream to downstream of the Xiaodao and Zhangzhen Rivers increased respectively from 0.8% and 7.3% to 48.8%. Then, the corresponding proportion of sewage sources decreased from 66.7% and 35.0% to 0.8%. This was in agreement with the proposition that there are higher proportions of surface water nitrate fertilizer sources in agricultural lands [48]. Before the rains, the proportion of chemical fertilizers and soil organic nitrogen in the Guangli River increased from 12.0% to 17.9%. Then the proportion of human/animal manure sources increased from 14.5% to 24.8%. Chemical fertilizers and soil organic nitrogen from the tributary (Yihong River) increased initially from 9.5% to 77.8% and then dropped to 25.4%. The proportion of nitrates from human/animal manure dropped from 61.5% to 21.0% and then again increased to 49.8%. The proportion changed after the river confluence was 57.4%. This was because the land-use type in the river basin changed from farmland to urban construction land, and was affected by point-source pollution from livestock farms [49].

Livestock breeding and household sewage discharge increased nitrate discharge in surface waters. The source of nitrate in Yellow River Farm Ditch changed with incoming rainfall. The ratio of chemical fertilizers to soil organic nitrogen dropped by 55.6% and that to human/animal manure increased by 52.0%. The proportion of nitrate in the surface water of the Yongfeng River that was from human/animal manure first increased from 55.5% to 74.1% and then later decreased to 64.2%. This was because the river first flowed through the livestock/poultry breeding area (increasing the proportion) and then later was diluted by precipitation, decreasing the proportion [49]. The proportions of chemical fertilizers, soil organic nitrogen, and human/animal manure in the surface water in the Yihong River decreased from 5.1%, 6.4%, and 7.8% to 0.3%, 0.4%, and 1.2%, respectively. The proportion of sewage sources (74.1–80.1%) was stable. Then the proportion of precipitation sources in the Guangli and Yihong Rivers increased from 0.5% to 27.0% and then to 24.0%. This was similar to what was observed for precipitation recharge to surface water by Castaldo et al. [50] and Zhang et al. [47]. Before the rains, the contribution rate of nitrate from surface water of the Xiaodao and Zhangzhen Rivers from chemical fertilizers, soil organic nitrogen, and human/animal manure decreased in trend. The proportion of nitrate in precipitation increased by 52.6 and 40.3% respectively from upstream to downstream, similar to the proportion of nitrate in precipitation entering the sea at 54.0% (±11.0%). R^2^ fit for the ratio of precipitation nitrate and seawater replenishment ratio in the water samples before and after the rains were 0.50 and 0.62, respectively. This suggested that seawater replenishment increased the nitrate ratio from rainfall. The area of water around the entrance to the sea is vast and is an offshore fishery breeding area. The proportions of nitrate from chemical fertilizers and soil organic nitrogen before the rains were 4.4% and 7.1%, respectively. Those after the rains were 5.0% and 7.9%, respectively. Precipitation did not significantly increase the proportion of nitrate sources in farmlands, but the proportion of manure sources increased from 12.6% to 21.8%. This was because heavy rainfall in the flood season increased drainage volume from fish farms [51].

### 3.4. Scientific Source Control of NO_3_^−^

Nitrate N is the main inorganic component of nitrogen that enters the water environment. In this study, human activities such as fertilizer application, domestic wastewater, and aquacultural waste were the main sources of NO_3_^−^. Specifically, it was caused by excessive use of nitrogen fertilizers in agricultural lands, rapid urbanization, population growth, and livestock/poultry breeding [53]. The Yellow River Delta is a middle-to-low-yield region where local farmers feel it necessary to use excessive chemical fertilizers for high crop yields. This does not necessarily meet nutrient requirements for plant growth. A precise economic benefit analysis of the salt-fertilization relationship was needed to guide farmers in daily farmland management and to reduce nutrient loss under rainfall and irrigation [54]. Conservation farming methods can minimize the impact on surface structures, strengthen infiltration, and lead to higher denitrification. Additionally, as the urban population increases, sewage discharge, and livestock breeding becomes the dominant sources of surface nitrate in water bodies. An increase in impervious surface areas produces more surface runoff and reduces N retention in watersheds [55]. Municipal treatment facilities including septic tanks, pit latrines, landfills, etc. contain sewage and leaking pipes that carry rich nutrients seep into the soil. Through mineralization and nitrification in the runoff, the nutrient is finally converted into nitrate that is easily soluble in water and therefore occurs in surface water [56]. There are large livestock/poultry breeding industries in the estuary delta and the typical model of livestock/poultry manure resource utilization can be vigorously pursued for combined planting and breeding. The clean reuse and discharge standards can enhance the integration of main non-point source pollutants (from livestock/poultry breeding systems) with ecological carrying capacity so that the absorption capacity threshold of coastal fragile zone ecosystems is not exceeded [57].

Riparian zones are vegetation buffer zones between farmlands and rivers. Understanding the nitrogen cycle in riparian zones and the related factors are necessary for feasible nitrogen emission management. Generally, there are three ways to alleviate nitrogen pollution through riparian zones: (1) Reducing surface runoff and related transportation of solutes and particles is an effective way to reduce the concentration of nitrogen adsorbed onto solute particles [48]. (2) Increasing the distance between farmland and the water body can extend the residence time of groundwater and thereby improve microbial degradation of dissolved nitrogen in groundwater [58]. (3) The absorption of nitrogen by vegetation during the growth period is critical in the nitrogen cycle [59]. The physical properties of buffer zones (such as width, slope, soil texture, and vegetation biomass) are the main factors driving nitrogen retention efficiency.

## 4. Conclusions

The estuary delta is located at the land-sea confluence. Nitrogen emission at the river outlet from the land into the sea has led to the deterioration of water quality in coastal areas. The concentration of nitrogen in rivers in agricultural basins will continue to increase. The relationship between surface water replenishment in the Yellow River Delta is affected by rain and irrigation from the Yellow River and other farmland water conservancy measures. From the source to the estuary, the proportion of recharge in Yellow River water decreased, but then recharge from groundwater and seawater increased. Rainfall changed the source of surface water replenishment. Surface water before rains replenished mainly groundwater (39.0%) and Yellow River water (33.6%). Water replenishment after rains was driven by water from the Yellow River (52.5%) and precipitation (28.8%). Rainfall diluted the concentration of nitrate N in the rivers in the study area but increased instantaneous nitrate N flux. After and before rains, nitrate N flux were respectively 862.59 and 499.16 g/s, an increase of 72.8%. There was a direct correlation between instantaneous nitrate N flux in the rivers after rainfall in the flood season and the river basin area. On this basis, findings were Zhimai River (493.51 g/s) > Guangli River (179.05 g/s) > Xiaodao River and Zhangzhen River (85.40 g/s) > Yellow River Farm Ditch (49.33 g/s). Nitrate N flux increased from upstream to downstream. Human activities such as excessive use of nitrogen fertilizers in agricultural areas, rapid urbanization, population growth, and livestock wastes were the main sources of nitrate in the study area, with total contributions of 60.8% before and 68.4% after rains. The adoption of an accurate management model for “salt content-fertilization-economic benefits” will guide local farmers in farmland management and strengthen the management of urban drainage and sewage. The promotion of a typical technical model for resource utilization of livestock manure was necessary for combined planting and breeding, and clean reuse, and up-to-standard discharge. This can result in integrated management of non-point source pollutant output, carrying capacity, and absorption capacity of fragile coastal ecosystems.

## Figures and Tables

**Table 1:** δ^15^N-NO_3_^−^ and δ^18^O-NO_3_^−^ values of potential NO_3_^−^ sources in surface water.

Source	Mean δ^15^N	SD δ^15^N	Mean δ^18^O	SD δ^18^O
fertilizer	−2.1	0.7	−4.1	2.7
sewage	17.4	3.9	6.1	1.6
precipitation	0.6	1.5	57.2	6.9
soil organic nitrogen	3.8	1.8	−2.7	4.4
manure	9.3	4.4	7.4	7.2

**Table 2:** Basic characteristics of the rivers in the study area.

Month	River	Length	Watershed Area	Average [NO_3_^−^-N]	Nitrate N Flux	δD-H_2_O	δ^18^O-H_2_O	δ^15^N-NO_3_^−^	δ^18^O-NO_3_^−^
		km	km^2^	mg/L	g/s	‰	‰	‰	‰
Before Rain	Yellow River Farm Ditch	18.80	100.00	0.70	34.18	−29.43	−2.93	−1.52	12.21
	Xiao Dao	27.50	120.80	0.95	21.45	−31.70	−3.14	6.46	24.86
	Zhang Zhen	28.00	140.00	1.47	21.45	−30.64	−3.47	6.75	28.68
	Yong Feng	33.80	200.00	1.93	3.87	−44.24	−5.44	8.19	11.70
	Guang Li	47.30	510.00	0.91	172.50	−50.36	−6.82	8.74	13.85
	Zhi Mai	135.00	3382.00	3.33	243.76	−53.83	−7.00	12.96	12.95
After Rain	Yellow River Farm Ditch	18.80	100.00	0.95	49.33	−51.35	−7.58	7.63	20.07
	Xiao Dao	27.50	120.80	2.16	85.40	−52.15	−7.89	5.89	20.76
	Zhang Zhen	28.00	140.00	3.09	85.40	−53.40	−8.03	3.67	22.83
	Yong Feng	33.80	200.00	1.80	30.38	−53.72	−7.69	11.29	20.04
	Guang Li	47.30	510.00	1.00	179.05	−59.14	−8.46	13.24	12.43
	Zhi Mai	135.00	3382.00	1.92	493.54	−71.43	−9.93	13.18	11.81

Note: the data of river length and drainage area are from Dongying water conservancy records [52].

## Data Availability

Not applicable.
